# Risk factors associated with postoperative complications after liver cancer resection surgery in western China

**DOI:** 10.1186/s12962-021-00318-z

**Published:** 2021-10-02

**Authors:** Yanjie Hu, Siyu Zeng, Lele Li, Yuanchen Fang, Xiaozhou He

**Affiliations:** 1 West China School of Nursing, West China Hospital, Sichuan University, No. 37#, Guoxue Road, Chengdu, China; 2 Business School, Sichuan University, No. 24 South Section 1, Yihuan Road, Chengdu, China; 3 School of Labor and Human Resources, Renmin University of China, No. 59 Zhongguancun Street, Beijing, China

**Keywords:** Logistic regression, Liver cancer surgery, Risk factor, Postoperative complications

## Abstract

**Objectives:**

Postoperative complications increase the workload of nursing staff as well as the financial and mental distress suffered by patients. The objective of this study is to identify clinical factors associated with postoperative complications after liver cancer resection surgery.

**Methods:**

Data from liver cancer resections occurring between January 1st, 2019 to December 31st, 2019 was collected from the Department of Liver Surgery in West China Hospital of Sichuan University. The Kruskal–Wallis test and logistic regression were used to perform single-factor analysis. Stepwise logistic regression was used for multivariate analysis. Models were established using R 4.0.2 software.

**Results:**

Based on data collected from 593 cases, the single-factor analysis determined that there were statistically significant differences in BMI, incision type, incision length, duration, incision range, and bleeding between cases that experienced complications within 30 days after surgery and those did not. Stepwise logistic regression models based on Kruskal–Wallis test and single-factor logistic regression determined that BMI, incision length, and duration were the primary factors causing complications after liver resection. The adjust OR of overweight patients and patients with obesity (stage 1) compared to low weight patients were 0.12 (95% CI:0.02–0.72) with p = 0.043 and 0.18 (95% CI:0.03–1.00) with p = 0.04, respectively. An increase of 1 cm in incision length increased the relative risk by 13%, while an increase of 10 min in surgical duration increased the relative risk by 15%.

**Conclusions:**

The risk of postoperative complications after liver resection can be significantly reduced by controlling factors such as bleeding, incision length, and duration of the surgery.

## Introduction

Liver cancer has the sixth-highest morbidity and the fourth highest mortality in the world. It has the fourth-highest morbidity and mortality in China [1]. According to GLOBOCAN2020, the annual number of new cases of liver cancer in the world reached 905,677 in 2020, of which most were from Asia [2]. The worldwide annual number of deaths due to liver cancer was 781,000. 120 million Chinese are carriers of HBV [3], and the Chinese government had made efforts on research, treatment and vaccination programs since 2007 [3]. The incidence of liver cancer in China is 2.51 times higher than that in the rest of the world, and the mortality rate is 2.54 times higher [4]. In particular, China has a large number of cases of hepatocellular carcinoma.Chinese government officially popularized hepatitis B vaccine for infants in 2015. Currently, the infection rate of hepatitis B virus in China is still quite high, which leads to a very high incidence of liver cancer in China. The National Cancer Center collected evidence and conducted a systematic evaluation of the economic burden of liver cancer in China between 1996 to 2015, it was concluded that the average direct medical costs incurred in hospitals of cases between 1996 to 2013 gradually increased, with a median of 1804.3 US dollars (541.45–10,916.7 US $) [5].The indirect economic burden, based on the human capital method, includes the lost working hours and early death of liver cancer patients as well as the lost working hours of caretakers. The median indirect economic burden was determined to be11353.82 US dollars (5536.99–25,813.09 US $). Through statistical analysis of the second-hand data, it can be concluded that suffering from liver cancer not only affects family income severely but also has high indirect costs. [6, 7].

At present, hepatectomy is the leading choice for radical treatment of liver cancer [8]. The European Association for the Study of the Liver (EASL) Clinical Practice Guidelines for Hepatocellular carcinoma [9] specify that radical resection is the first choice for the treatment of liver cancer. However, radical resection of liver cancer is a lengthy and complex abdominal operation that is often accompanied by severe trauma. Anatomically, the liver is close to the diaphragm, its vascular structure is complex, and the intrahepatic bile duct is closely accompanied by blood vessels. This complex structure leads to a high risk of bleeding during liver surgery and a high incidence of postoperative complications such as bile leakage, liver failure, ascites, and pulmonary infection. Furthermore, owing to the adhesion between the tumor and diaphragm and the separation of the diaphragm during the operation, it is easy to incur pleural effusions and pulmonary infection. The surgical incision of the liver is located under the costal margin, which can be traumatic, and the degree of postoperative pain is high, impeding the postoperative activities of the patient such as taking deep breaths, coughing, and expectoration. These complications affect the postoperative recovery of patients, threaten the physical and mental health of patients, prolong their stay in the hospital, and might even result in further surgery or death. Consequently, determining the factors influencing postoperative complications in hepatectomy patients is an important research topic in the field of liver cancer treatment.

Logistic regression model is one of the common methods of machine learning (machine learning, ML), which can find and identify patterns and relationships between variables from complex data sets, identify risk factors and provide a coefficient of the influence of a factor on the outcome variable [10]. Logistic regression is an effective way to study the risk factor of postoperative complications after liver cancer resection surgery, but the relative research are limited. In addition, what is most worthy of integration is that based on the "patient-centered" clinical health service model, combined with the accumulation of research in the field of diagnosis and treatment of liver cancer, it is innovatively proposed to explore the risk factors of complications after radical resection of liver cancer from all aspects of patients' demography-related factors, disease-related factors and operation-related factors, and pay attention to the full-cycle and full-process health care of patients [11, 12]. In this study,based on the “patient-centered” clinical health service model combined with data collected from research in the field of diagnosis and treatment of liver cancer, we built a logistic regression model to explore the risk factors of complications after radical resection of liver cancer arising from patients' demography-related factors, disease-related factors, and operation-related factors.

## Materials and methods

### Study population

The relevant data of patients who underwent hepatectomies between January 1, 2019, and December 31, 2019, were collected from the Department of Liver Surgery of West China Hospital of Sichuan University.

Inclusion criteria: (1) pathologic diagnosis was hepatocellular carcinoma; (2)18–65 years old; (3) ASA scores I to III; (4) informed consent.

Exclusion criteria: (1) patients with other malignant tumors; (2) patients with metabolic syndrome. ① Patients with hyperglycemia, fasting blood glucose (FPG) ≥ 6.1 mmol/L (110 mg/dl) and/or 2hPG ≥ 7.8 mmol/L (140 mg/dl), and/or those who had been diagnosed and treated with diabetes.② Hypertensive patients with systolic blood pressure/diastolic blood pressure ≥ 140 pm 90 mmHg, and/or those who had been diagnosed and treated for hypertension. ③ Dyslipidemia, fasting triglyceride ≥ 1.7 mmol/L (150 mg/dl), and/or fasting blood HDL-C < 0.9 mmol/L (35 mg/dl) (male), < 1.0 mmol/L (39 mg/dl) (female). Patients with 3 or all of the above 4 components can be diagnosed with metabolic syndrome (*Diagnostic criteria recommended by The Chinese Diabetes Society*).

**Fig. 1:**
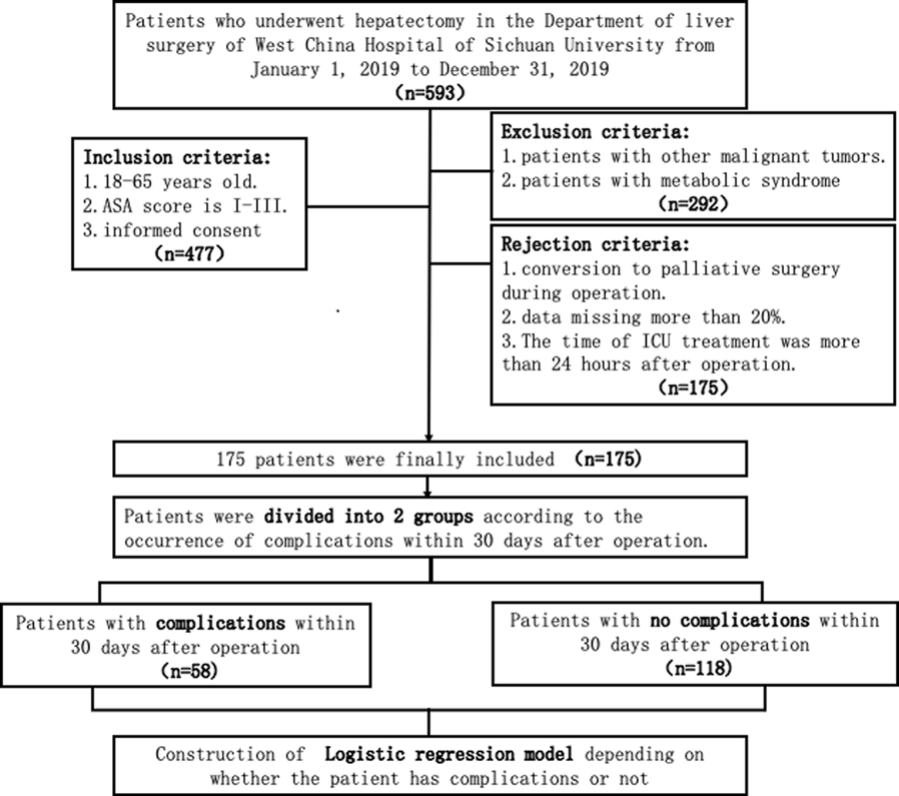
Inclusion criteria flowchart of patients

Rejection criteria: (1) conversion to palliative surgery during surgery; (2) more than 20% data missing; (3) ICU admission being more than 24 h after surgery. The inclusion criteria flowchart of patients are seen in Fig.1 .

### Data collection

The collected data were cleaned, and patients who were transferred to palliative surgery or the ICU after the operation were excluded. After deleting missing values and abnormal values, data from 175 patients were obtained, including sex, age, diagnosis, height, weight, tumor diameter, tumor number, and other parameters.

Based on expert knowledge, the data we collected included 14 independent variables representing potential influencing factors for postoperative complications, which could be divided into descriptive information (namely, age, sex, and body mass index (BMI)), disease information (namely, Ishak score, tumor diameter, tumor number, invasion of capsule, tumor thrombus, and tissue differentiation) and surgical information (namely, incision type, incision length, operation time, resection range, and blood loss). Specific data variables, sample assignments, and descriptive information are listed in Table 1. The original data we collected were then cleaned and sorted.

**Table 1:** Baseline patient characteristics in the Complications group and Non-Complications group (N = 175)

Variables	Variable category (assignment)	Sample size (N %)	Complications group (N = 58)	Non-complications group (N = 116)	Kruskal–Wallis chi-squared	P-value
Demographic information						
Age	18–45:1	50 (28.57%)	14 (24.14%)	36 (30.77%)	0.83077	0.3621
	45–65:2	125 (71.42%)	44 (75.86%)	81 (69.23%)		
Gender	Male (1)	144 (82.29%)	48 (82.76%)	96 (82.05%)	0.013234	0.9084
	Female (2)	31 (19.61%)	10 (17.24%)	21 (17.95%)		
BMI (kg/m^2^)	< 18.5 (1)	13 (7.43%)	4 (6.90%)	9 (7.69%)	10.053	0.03954
	18.5–23 (2)	88 (50.29%)	38 (65.52%)	50 (42.74%)		
	23–25 (3)	39 (22.29%)	7 (12.07%)	32 (27.35%)		
	25–30 (4)	33 (18.86%)	7 (15.51%)	24 (20.51%)		
	> 30 (5)	2 (1.14%)	0 (0%)	2 (1.71%)		
Disease information						
ISHAK	Continuous variable ($mean$ $\pm SD$)	4.94 $\pm 1.34$	4.86 $\pm 1.22$	4.97 $\pm 1.09$	1.9552	0.744
Tumor diameter	Continuous variable ($mean\pm SD$)	5.69 $\pm 3.40$	6.58 $\pm 3.49$	5.26 $\pm 3.28$	70.943	0.1577
Tumor number	1 (1)	145 (82.86%)	46 (79.31%)	99 (84.61%)	5.187	0.3935
	2 (2)	16 (9.14%)	4 (6.90%)	12 (10.23%)		
	3 (3)	7 (4.00%)	4 (6.90%)	3 (2.56%)		
	4 (4)	4 (2.29%)	2 (3.45%)	2 (1.70%)		
	5 (5)	2 (1.14%)	1 (1.72%)	1 (0.85%)		
	6 (6)	1 (0.57%)	1 (1.72%)	0 (0%)		
Invade the capsule or not	No (0)	84 (48.00%)	24 (41.37%)	60 (51.28%)	1.5148	0.2184
	Yes (1)	91 (52.00%)	34 (58.62%)	57 (48.71%)		
With cancer thrombus	No (0)	133 (76.00%)	41 (70.69%)	92 (78.63%)	1.3336	0.2482
	Yes (1)	42 (24.00%)	17 (29.31%)	25 (21.37%)		
Tissue differentiation	High differentiation (1)	4 (2.29%)	1 (1.72%)	3 (2.56%)	2.0111	0.3658
	Middle differentiation (2)	152 (86.86)	48 (82.76%)	104 (88.89%)		
	Low differentiation (3)	19 (10.85%)	9 (15.51%)	10 (8.55%)		
Surgical information						
Incision type	Kocher’s incision (1)	129 (73.71%)	51 (87.93%)	78 (66.67%)	8.9975	0.002704
	Abdominal incision (2)	46 (26.28%)	7 (12.07%)	39 (33.33%)		
Incision length	Continuous variable ($\mathrm{mean}\pm \mathrm{SD}$)	23.31 $\pm 4.61$	24.78 $\pm 3.67$	22.58 $\pm 4.87$	37.703	0.04944
Duration	Continuous variable ($mean$ $\pm SD$)	201.04 $\pm 58.$	234.1$\pm 59.47$	184.64$\pm 51.44$	64.607	0.04017
Incision range	One liver segment (1)	37 (21.14%)	5 (8.62%)	32 (27.35%)	16.294	0.0009869
	Two liver segments (2)	55 (31.42%)	16 (27.59%)	39 (33.33%)		
	Three liver segments (3)	44 (25.14%)	15 (25.86%)	29 (24.78%)		
	Four liver segments (4)	39 (22.28%)	22 (37.93%)	17 (14.53%)		
Bleeding	Continuous variable ($mean\pm SD$)	380$\pm 307.82$	516.72 $\pm 382.87$	312.22 $\pm 236.90$	34.274	0.02433
Outcome variables						
Complications	No (0)	117 (66.48%)				
	Yes (1)	58 (33.52%)				

In the study, postoperative complication included bile leakage (defined as the serum bilirubin continues to appear in the drain fluid for more than 7 days after surgery), incision site infection (the incision has purulent secretion and positive bacterial culture, or infection such as redness, swelling, heat, pain and so on), pulmonary infection (sputum culture positive or lung auscultation indicating decreased respiratory sounds or wet rales, chest CT and X-ray indicating pulmonary inflammation), abdominal infection (purulent secretion from abdominal drainage tube, purulent secretion from abdominal puncture, detection of pathogenic bacteria in secretion, or abdominal abscess confirmed by abdominal B-ultrasound), urinary tract infection (frequent urination, urgent urination, urinary pain and other symptoms of urinary tract infection, urine bacterial culture is positive), ascites, pleural effusion, intestinal ischemia, gastrointestinal bleeding, cholangitis, portal vein thrombus, ileus, acute colitis, acute renal dysfunction.

### Statistical analysis

Kruskal–Wallis test is used for single-factor analysis [12]. For categorical variables, chi-square tests are used to calculate differences between two independent groups. Logistic regression model, a popular machine learning method, is used to find and identify patterns and relationships between variables from complex data sets, identify risk factors, and provide a coefficient of the influence of a factor on the outcome variable [13]. Based on earlier studies, the logistic regression model is used to identify the factors influencing complications after hepatectomy, in order to provide references for the prevention of complications after hepatectomy. First, the single-factor analysis was conducted using the Kruskal–Wallis test, which was used to compare all the potential variables as there are more than two groups with a non-normal distribution. The statistically significant variables were considered as risk factors according to the Kruskal–Wallis test. Then, to describe the specific numerical relationship between the probability of the complications and the risk factors as well as to predict the occurrence of complications, a multivariate stepwise logistic regression model was established based on the risk factors using the “glm package” in R 4.0.2 to explore the influencing factors of complications after hepatectomy. A value of p < 0.05 was considered statistically significant. R 4.0.2 software (open source software) was used for data analysis and modeling.

## Results

**Fig. 2:**
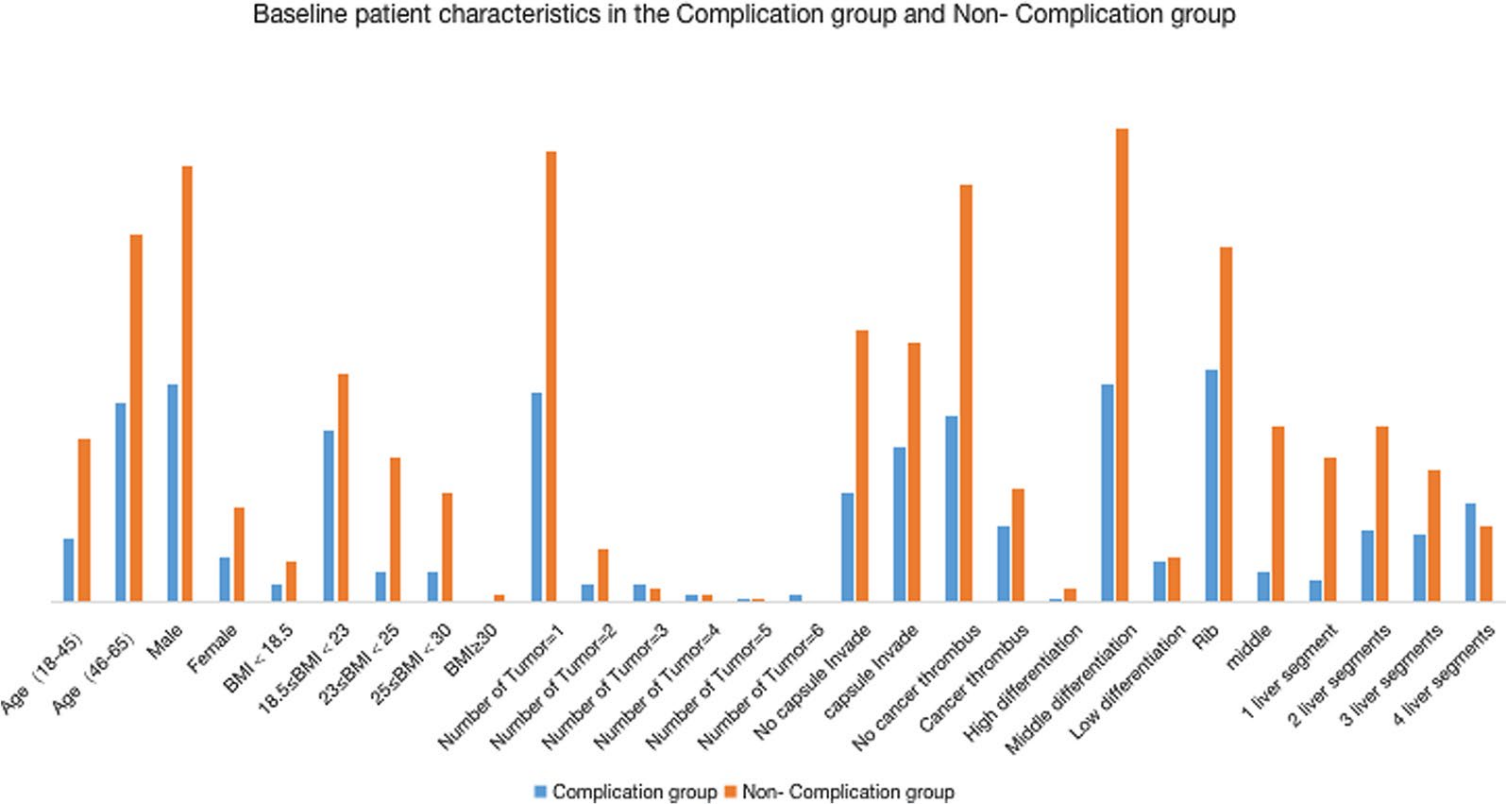
Histograms of patients’ information in the Complications group and Non-Complications group

Data from 477 patients who underwent liver cancer surgery between January 1, 2019, and December 31, 2019, were analyzed. Data from 185 cases (38.70%) were excluded because those patients had other malignant tumors or metabolic syndrome. Data from 117 cases (40.01%) were rejected because those patients converted to palliative surgery during the operation, the data had more than 20% missing entries, or the time of ICU treatment was more than 24 h after surgery. Of the 175 cases included in this study, 59 patients (33.71%) experienced complications within 30 days after surgery; these cases constitute the ‘Complications’ group. The other 116 patients (66.29%) were classified into the ‘no complications within 30 days after operation’ group, also called the control group or the ‘Non-Complications’ group. Patients’ information such as age, sex, body mass index (BMI)), tumor number, invasion of capsule, tumor thrombus, tissue differentiation, incision type, and resection range in the Complications group and Non-Complications group is shown in Fig. 2.

As can be seen in Table 1, BMI, incision type, incision length, duration, excision range, and bleeding differed significantly between the Complications and Non-Complications groups, with p-values < 0.05, respectively. The other factors were not statistically significant.

**Fig. 3:**
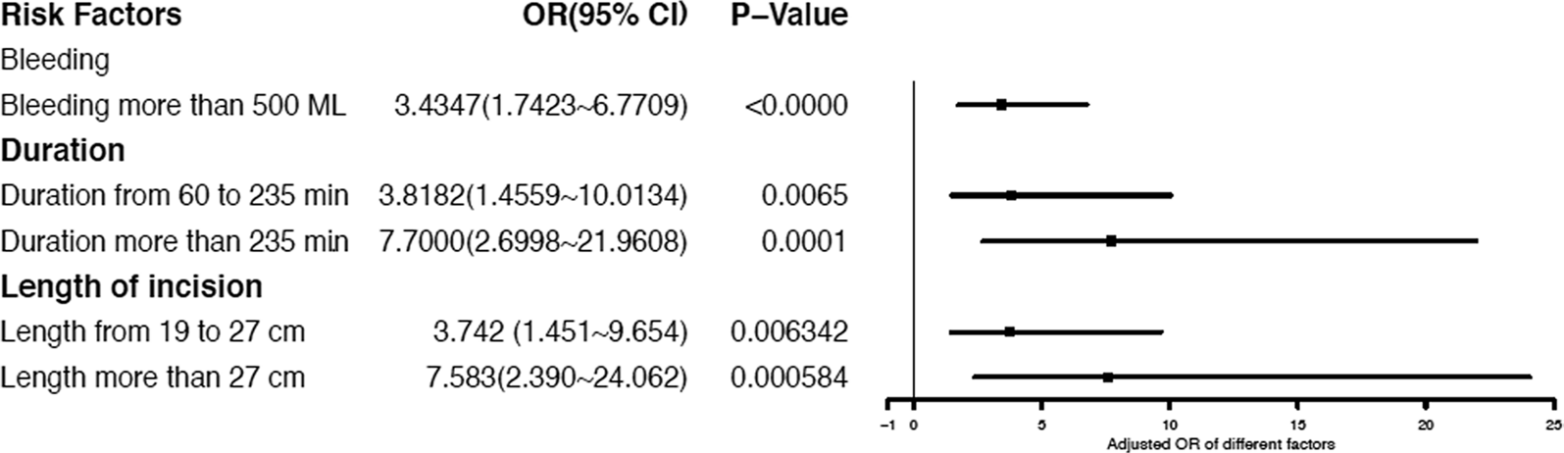
Forest map of OR value (95%CI) and p value of risk factors from single-factor

As presented in Appendix 1, we also used single-factor logistic regression to identify the major influencing factors. Seven factors are shown to be statistically significant in the single-factor analysis: type of incision, incision length, BMI, tumor size, duration, range of incision, and bleeding. The forest map of OR values (95% CI) and p-values of risk factors from single-factor logistic regression analysis are shown in Fig. 3.

**Fig. 4:**
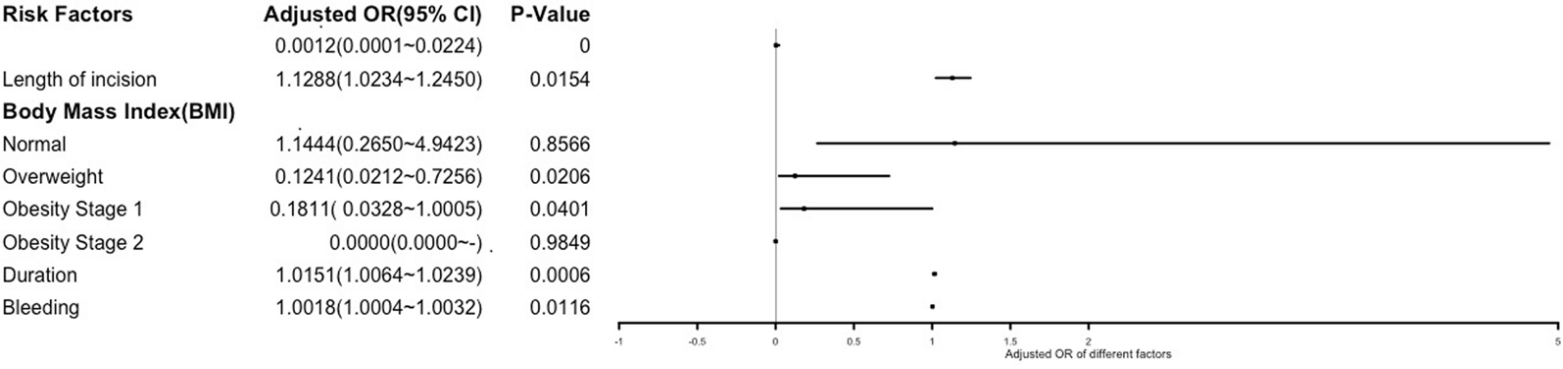
Forest Plot of Multiple Stepwise Logistic Regression Analysis

We constructed stepwise logistic regression models based on Kruskal–Wallis test and single-factor logistic regression. We found that although the variables screened by the two methods were different, the output results of the stepwise logistic regression model were consistent. This indicates that our model is stable. As presented in Table 2, incision length, BMI, duration, and bleeding were the primary factors related to complications after liver resection surgery. The four variables’ Akaike information criterion (AIC) was 178.23, the effect was the best, and all the variables were statistically significant; the results are presented in Table 2. The statistically significant variables’ OR values (95% CI) and p-values are given in the forest map in Fig. 4.

**Table 2:** Multiple stepwise logistic regression analysis of factors related to complications

Risk factors	SE (standard eror)	Z-score	Adjusted OR (95% CI)	p-value
Coefficient	1.5066	− 4.4805	0.0012 (0.0001–0.0224)	≤ 0.0001
Incision length	0.0500	2.4221	1.1288 (1.0234–1.2450)	0.0154
Body Mass Index (BMI)				
Normal (18.5 ≤ BMI < 23)	0.7464	0.1807	1.1444 (0.2650–4.9423)	0.8566
Overweight (23 ≤ BMI < 25)	0.9011	− 2.3160	0.1241 (0.0212–0.7256)	0.0206
Obesity (Stage 1) (25 ≤ BMI < 30)	0.8722	− 1.9594	0.1811 (0.0328–1.0005)	0.0401
Obesity (Stage 2) (BMI > = 30)	890.9808	− 0.0189	0.0000 (0.0000)	0.9849
Duration	0.0044	3.4158	1.0151 (1.0064–1.0239)	0.0006
Bleeding	0.0007	2.5254	1.0018 (1.0004–1.0032)	0.0116

BMI : refers to Asian BMI classification

Statistically significant if p-value ≤ 0.05

The multiple logistic regression analyses revealed that patients who had longer lengths of incision were significantly more likely to experience complications compared to those who had shorter incision lengths (adjusted OR: 1.13 (1.02–1.25), p = 0.0154). Based on our research, low-weight patients were more likely to suffer complications after liver resection surgery than any other groups; their chances were 8.06 times higher than the overweight patients and 5.52 times higher than the patients with obesity (stage 1). The adjusted OR of the overweight patients and obesity (stage 1) patients groups compared with the low-weight group were 0.12 (95% CI: 0.02–0.72), p = 0.043 and 0.18 (95% CI:0.03–1.00), p = 0.04, respectively.

Duration and bleeding also show significant statistical relationships with the Complications group. They are continuous variables, measured in minutes and milliliters, respectively. After multi-factor analysis determined that they are major influencing factors, we used the quartile of this batch of data as the demarcation line. Details are given in Appendix 2. After analysis of the blood loss, the upper quartile 500 ml was taken to divide the blood loss into two categories: low blood loss grade 1 (blood loss < 500 ml) and high blood loss grade 2 (blood loss < 500 ml). Our research indicates that high blood loss grade 2 patients were 3.43 times more likely to suffer complications after liver resection surgery than low blood loss grade 1 groups, (bleeding > 500 ml, adjusted OR: 3.56 (95% CI:1.74–6.77), p = 0.00036). Patients whose surgeries had durations of 160–235 min were 3.8 times likelier than those whose surgeries had a duration of fewer than 160 min to experience complications after liver resection surgery (160 ≤ duration < 235, adjusted OR: 3.82 (95% CI:1.46–10.01), p = 0.0065), and those whose duration of surgery was more than 235 min were 7.7 times likelier than those whose surgeries lasted fewer than 160 min (duration > 235, adjusted OR: 7.70 (95% CI:2.70–21.96), p = 0.00014).

## Discussion

Postoperative complications after liver cancer resection surgery have attracted the attention of many researchers, and many studies have found out risk factors (11,14–16). Logistic regression, as the most classical algorithm of machine learning, is an effective way to predict the risk factors of disease and the probability of disease occurrence according to the risk factors (10,12), but the related researches are quite limited. No researchers in China use this machine learning way to study the risk factor of postoperative complications after liver cancer resection surgery, this study has fill this gap.

Of 175 cases who underwent liver cancer surgery, 59 had complications within 30 days after surgery (33.71%). Most of the cases that experienced complications within 30 days after surgery were managed using conservative treatment (58 cases, 98.31%) including complete bed rest, antiemetics, cough suppressants, cooling treatment, anti-infective therapy, liver protection therapy, and humoral management therapy for 2–5 days. Conservative treatment failed in only 1 out of 59 cases, which was then subjected to another operation within 30 days after the first operation. A recent study found that the incidence of postoperative complications after liver resection was 30%–50%. It was consistent with the results of our study 33.71% [11, 14]. In this study, 14 pre-operative and post-operative initial variables were included in the analysis based on expert opinions. First, 7 independent variables with p < 0.05 were found as the statistically significant main factors among the 14 factors based on single-factor regression analysis and Kruskal–Wallis test. These were BMI, incision type, incision length, duration, incision range, tumor size, and bleeding. Finally, stepwise regression was used to screen the secondary modeling variables based on the minimum AIC. Four variables (incision length, BMI, duration, bleeding) that make the model most interpretable were selected to be entered into the final model, with a minimum AIC of 178.23. After experts in liver surgery examined and analyzed the results, corresponding clinical suggestions were given.

In the descriptive statistics, the results showed that the preoperative BMI level in patients with liver cancer was negatively correlated with the risk of postoperative complications, which was inconsistent with the previous study. For low-weight patients, as classified via BMI, a decrease in BMI was associated with an increased risk of complications within 30 days after surgery. This was especially the case for low-weight patients, who were 8.06 and 5.52 times likelier to experience a risk of complications than Overweight patients (23 ≤ BMI < 25) and Obesity (Stage 1) patients (25 ≤ BMI < 30), respectively. The comparison in the normal weight group did not show any statistical significance. The ORs of overweight patients and patients with obesity stage 1 compared with low weight patients were 0.12 (95% CI:0.02–0.72) with p = 0.043 and 0.18 (95% CI:0.03–1.00) with p = 0.04, respectively.

Based on further analysis of the BMI data of liver cancer patients in this study, it was found that 74.36% of liver cancer patients were malnourished before surgery, and the level of BMI index was low (mean 22.51, quartile 24.26). A possible reason for this was that patients with liver cancer have a high incidence of preoperative malnutrition caused by loss of appetite, disturbance of lipid metabolism and protein synthesis, and tumor cachexia. Low-weight patients show increased ascites, pleural effusions due to the lack of energy needed by the body, and damaged physiological functions, reducing their immunity and even causing systemic organ failure. Therefore, our results suggested that clinical doctors and nurses should pay attention to the preoperative nutritional status of patients with liver cancer, and routinely evaluate the nutritional status of patients with liver cancer who are scheduled to undergo hepatectomy. Patients with malnutrition should be treated with timely nutritional support in a scientific manner to improve their nutritional status, improve their tolerance to surgery, and reduce the risk of postoperative complications [15].

The results of surgical information were consistent with previous studies [16, 17]. The operation time and incision length were positively correlated with the risk of complications. This is because operation time and incision length are indicators of the degree of trauma caused by the operation. The higher the operation time and longer the incision length, the more severe the surgical trauma, the stronger the stress reaction, hyperimmunity, and inflammation of the body, and consequently, the higher the risk of postoperative complications. From the point of view of enhanced recovery after surgery (ERAS) based on the concept of evidence-based medicine, perioperative diagnosis and treatment programs should be optimized as much as possible, so as to avoid diagnoses and treatment measures that are not beneficial (or even potentially harmful) to the body and minimize the stress level of patients in order to achieve rapid and safe recovery [18]. Therefore, it is suggested that the clinical surgeon should create a multidisciplinary expert group to discuss the operation plan before the operation, and an accurate resection plan should be designed by multidisciplinary experts such as liver surgeons, anesthesiologists, and vascular surgeons, while also taking into consideration factors such as tumor size, location, and Child–Pugh score. It was also suggested that clinical medical staff should adopt the concept of ERAS during surgery, integrate medical and nursing care to ensure high quality, optimize the process of operation preparation, communicate effectively with patients and their families, and reduce the number of times that turntables are used in order to shorten the duration of surgery as much as possible, so as to decrease the risk of postoperative complications.

The results also showed that the amount of intraoperative bleeding was a negative indicator of postoperative complications risk, (OR: 1.0018 (95% CI:1.0004–1.0032), p-value = 0.0116), which was consistent with the results of previous studies [19]. In order to further explore the clinical significance of intraoperative bleeding volume, we refer to previous literature [20, 21] and use 500 ml as the critical value to classify the data of intraoperative blood loss. Further analysis of the data showed that in the two categories of intraoperative blood loss, the risk of complications in patients with a blood loss of more than 500 ml was 3.32 times higher than that in patients with blood loss less than 500 ml. The reason may be that intraoperative bleeding higher than 500 ml can cause liver and intestinal ischemia, resulting in damage to the structure and function of hepatocytes. Furthermore, intestinal mucosa can easily cause intestinal flora translocation due to ischemia and hypoxia, leading to infectious complications [22]. Therefore, it was suggested that surgical teams should follow the principle and concept of ERAS during surgery, accurately remove the focus, refine the operation, reduce intraoperative bleeding, and endeavor to control the amount of bleeding to keep it below 500 ml.

It was worth noting that in the disease information, the single-factor model showed that there was a significant statistical relationship between the size of the tumor and complications, but in the selection of the multifactor stepwise regression model, the explanatory effect of this variable on the model was minor and was eliminated, so there were no related variables of disease information in the final model. It was suggested that the disease factors of patients with liver cancer can provide a reference for the risk prediction of postoperative complications to a certain extent, but the results tend to suggest that clinical medical staff should pay more attention to the functional status of patients undergoing hepatectomy and the quality of the operation process. It also explains and emphasizes the importance of perioperative nutrition management, control and management of complications, and accelerated surgical process quality as outlined by the concept of ERAS.

To summarize, our study helped identify 14 factors that may lead to complications and collected the relevant data from 21,996 fields from 175 patients. Through univariate analysis and stepwise regression, four variables with significant statistical significance were selected to construct a logistic regression model. Identifying the risk factors of postoperative complications in patients with hepatectomy is helpful for medical staff to intervene in the relevant indicators before surgery and reduce the risk of postoperative complications. The model is also of considerable significance as it enables the early detection of postoperative complications. It can help surgeons to identify patients with a potentially high risk of postoperative complications and take pre-emptive measures in postoperative nursing.

However, there are some limitations in the study owing to the lack of data information such as patient behavior (e.g. smoking) and patient physical status (e.g. comorbidity). In the future, our research team will conduct a multicentered, high-quality randomized controlled study on the perioperative risk factors of radical resection of liver cancer using a larger sample size. We will also explore the accurate prediction of the risk of postoperative complications with the help of advanced data analysis techniques such as machine learning. Comprehensive studies that include data such as patient behavior information and disease history will also be conducted.

## Conclusion

Length of incision, BMI, duration, and bleeding were the main factors related to complications after liver resection surgery. Low-weight patients whose BMI was less than 18.5 had 8.06 and 5.52 times the risk of complications after liver resection as compared to overweight and obesity (stage 1) patients, respectively. An increase of 1 cm in incision length increased the relative risk by 13%, while an increase of 10 min in surgical duration increased the relative risk by 15%. Further research is needed to gain more insight into the causes of postoperative complications after hepatectomies.

## Data Availability

Data and materials are from the whole experiment approved by the Institutional Review Board (IRB) of West China Hospital in Sichuan University on the 30st of November 2016.

## Appendix 1

See Table 3.

**Table 3:** Single logistic regression analysis of factors related to postoperative complications

Variables	Coefficient	Standard error	*Z*	*P*
Incision type (2)	− 1.4798	0.5286	− 2.799	0.00512
Incision length	0.12167	0.04632	2.627	0.00862
BMI	− 0.16907	0.07151	− 2.364	0.0181
Tumor size	0.16963	0.06209	2.732	0.00629
Duration	0.019171	0.004306	4.453	8.48e − 06
Incision range (2)	0.5173	0.6676	0.775	0.438473
Incision range (3)	1.4971	0.6647	2.252	0.024310
Incision range (4)	2.4024	0.6688	3.592	0.000328
Blood loss	0.0022275	0.0005908	3.770	0.000163

## Appendix 2

See Table 4.

**Table 4:** Further discussion of the duration and bleeding related to complications (single logistic regression analysis)

Risk factors	SE (standard error)	Z-score	OR (95% CI)	P-value
Bleeding				
High blood loss grade 2 (blood loss > 500 ml)	0.3463	3.5633	3.4347 (1.7423–6.7709)	$\le$ 0.0001
Duration				
Group 1 (160 min < duration ≤ 235 min)	0.4919	2.7235	3.8182 (1.4559–10.0134)	0.0065
Group 2 (duration > 235 min)	0.5347	3.8173	7.7000 (2.6998–21.9608)	0.0001

500 ml is based on the quartile and the doctor's recommendation

160 min was the quartile of duration and 235 min was three quartiles
